# Examining changes in pain interference via pandemic-induced isolation among patients receiving medication for opioid use disorder: a secondary data analysis

**DOI:** 10.1186/s12889-024-20077-9

**Published:** 2024-09-27

**Authors:** Tessa Frohe, Tim Janssen, Bryan R. Garner, Sara J. Becker

**Affiliations:** 1 Department of Psychiatry & Behavioral Sciences, School of Medicine, University of Washington, 1959 NE Pacific ST, Box 357238, Seattle, WA 98195-7238 USA; 2 Center for Alcohol and Addiction Studies, Brown University School of Public Health, Providence, RI USA; 3 Department of Internal Medicine, Ohio State University, The Ohio State University College of Medicine, 2050 Kenny Road, Columbus, 43221 USA; 4 Center for Dissemination and Implementation Science, Northwestern University Feinberg School of Medicine, 633 North St Clair, Suite 2000, Chicago, IL USA

**Keywords:** Pain, Social isolation, Substance use, Medication for opioid use disorder, Opioid treatment program, Pandemic

## Abstract

**Background:**

Early in the pandemic, the United States population experienced a sharp rise in the prevalence rates of opioid use, social isolation, and pain interference. Given the high rates of pain reported by patients on medication for opioid use disorder (MOUD), the pandemic presented a unique opportunity to disentangle the relationship between opioid use, pain, and social isolation in this high-risk population. We tested the hypothesis that pandemic-induced isolation would partially mediate change in pain interference levels experienced by patients on MOUD, even when controlling for baseline opioid use. Such work can inform the development of targeted interventions for a vulnerable, underserved population.

**Methods:**

Analyses used data from a cluster randomized trial (*N* = 188) of patients on MOUD across eight opioid treatment programs. As part of the parent trial, participants provided pre-pandemic data on pain interference, opioid use, and socio-demographic variables. Research staff re-contacted participants between May and June 2020 and 133 participants (71% response rate) consented to complete a supplemental survey that assessed pandemic-induced isolation. Participants then completed a follow-up interview during the pandemic that again assessed pain interference and opioid use. A path model assessed whether pre-pandemic pain interference had an indirect effect on pain interference during the pandemic via pandemic-induced isolation.

**Results:**

Consistent with hypotheses, we found evidence that pandemic-induced isolation partially mediated change in pain interference levels among MOUD patients during the pandemic. Higher levels of pre-pandemic pain interference and opioid use were both significantly associated with higher levels of pandemic-induced isolation. In addition, pre-pandemic pain interference was significantly related to levels of pain interference during the pandemic, and these pain levels were partially explained by the level of pandemic-induced isolation reported.

**Conclusions:**

Patients on MOUD with higher use of opioids and higher rates of pain pre-pandemic were more likely to report feeling isolated during COVID-related social distancing and this, in turn, partially explained changes in levels of pain interference. These results highlight social isolation as a key risk factor for patients on MOUD and suggest that interventions promoting social connection could be associated with reduced pain interference, which in turn could improve patient quality of life.

**Trial registration:**

NCT03931174 (Registered 04/30/2019).

## Background

The COVID-19 pandemic started while America was in the throes of another public health emergency associated with the rise in opioid-related overdose deaths. As these two public health crises collided, the COVID-19 crisis created a range of new risk factors for patients on medication for opioid use disorder (MOUD), including higher-than-average rates of COVID-19 infection, job insecurity, closure of opioid treatment programs that dispense medication, and social distancing or shelter-in-place orders limiting chance of bystander overdose rescue [1–5]. This constellation of risk factors coincided with an exponential acceleration in overdose related deaths, with the largest spike occurring from March 2020 to May 2020 [6], during the beginning of the US quarantine lockdowns. Early COVID research findings during this time have indicated increased opioid and other drug use [7–11] accompanied by a sharp rise in rates of social isolation. These findings highlight the critical importance of understanding the effects of social isolation on persons with opioid use disorder (OUD).

One potential pathway through which social isolation may affect those with OUD is through its relationship with pain. Pain is a well-established predictor of OUD [12–15], is common among patients on MOUD [16], and is a predictor for worse treatment outcomes among patients receiving MOUD [17–19]. Longitudinal studies have shown that social isolation predicts the extent to which pain interferes with usual activities (commonly referred to as “pain interference”), even when controlling for a range of covariates. Epidemiological and neurological data suggest that the relationship between pain interference and social isolation is likely bidirectional [20–22], meaning that heightened pain interference can cause increases in social isolation, and increases in social isolation can cause increases in subsequent pain interference. Further, data from prior studies suggests pain interference may worsen substance use disorder (SUD) symptoms through negative affect [23], making for a dangerous, cyclical relationship among pain interference, substance use, and negative affect that may be attributable to social isolation. During the early pandemic, rates of social isolation and pain interference both increased dramatically [24–26], highlighting a rare opportunity to disentangle the relationship between pain interference and social isolation among patients receiving MOUD.

Karos and colleagues [27] hypothesized that the social isolation associated with the COVID pandemic was likely to disproportionately affect individuals with chronic pain, and led to exacerbation of pain symptoms. A cross-sectional study among the general population in Japan provided partial support for this hypothesis: the investigators found that perceived severity of social isolation during the COVID-19 lockdown was positively associated with both the prevalence of pain and the intensity of pain reported [26]. Similarly, a study of 150 patients who identified as having chronic pain in Massachusetts found that patients self-reported an increase in pain interference within the first few months following social distancing [28].

Given the higher prevalence rates of pain interference among patients on MOUD (approximately 40% report chronic interfering pain) [29], it is important to examine the ways that social distancing may have impacted patients on MOUD. The current study sought to examine whether pandemic-induced isolation statistically mediated the effect of pre-pandemic pain interference on subsequent pain interference during the pandemic, controlling for pre-pandemic opioid use. We hypothesized that pain interference levels prior to the pandemic would directly predict higher levels of pandemic-induced isolation, which would in turn would directly predict greater pain interference at subsequent follow up appointments. We further hypothesized that pain interference levels prior to the pandemic would have an indirect effect on pain interference during the pandemic via pandemic-induced isolation.

## Methods

### Parent study

This is a secondary data analysis from a larger, ongoing, parent study called Project [Masked] (Masked for Review). Participants provided informed consent prior to completing any study procedures. The study was approved by the involved university Institutional Review Board (IRB). Project [Masked] was a cluster-randomized type 3 implementation-effectiveness hybrid trial (grant/clinicaltrials.gov Masked [04/30/2019]) that is focused on testing two implementation strategies for helping opioid treatment programs (OTPs) to implement contingency management, an evidence-based behavioral intervention in which patients earn prizes for meeting treatment goals [30–33]. When COVID-19 and social distancing regulations began in March 2020, Project [Masked] had enrolled 188 patient participants from eight OTPs (Masked citation). Participants had been enrolled on a rolling basis over a six-month enrollment period (August 2019 – January 2020): to qualify, each participant had to report being inducted on MOUD within 30 days of their enrollment date. Upon enrollment, each participant completed a baseline survey that assessed their substance use and pain (see Measures). The same survey was repeated at 3-, 6-, and 9-month follow-up assessments.

### COVID-19 impact assessment

In accordance with IRB-approved procedures, enrolled participants were invited in the early months of the pandemic (May-July 2020) to complete a supplementary survey on COVID-19-related impacts. After providing informed consent for this supplemental aspect of the main study, participants completed the Epidemic-Pandemic Impact Inventory (i.e., EPII Survey); a scale consisting of 92 binary (yes/no) items that inventory the impact of COVID across 10 life domains: employment, interpersonal conflict, social isolation, economic, emotional health, substance use, physical health, quarantining and physical distancing, infection exposure, and caretaking. The survey took about 15–20 min to complete, and patients received $20, which was added to the rechargeable gift card they had already received as part of Project [Masked]. Participants then completed routine Project [Masked] follow-up assessments.

For the current study, each participant’s first follow-up assessment to follow the EPII assessment was used to calculate the effect of COVID-related impacts on subsequent pain interference. These follow-up surveys were conducted between May 2020 and January 2021. The analysis examined participant data collected across three timepoints: Timepoint 1 (pre-pandemic, August 2019 – March 2020); Timepoint 2 (during-pandemic, May 2020 – July 2020); Timepoint 3 (during pandemic, May 2020 – January 2021). Of note, Timepoint 1 data collection occurred prior to MOUD induction whereas Timepoint 2 and 3 both occurred post MOUD induction. Because recruitment for this study was on a rolling basis and all participants completed Timepoint 2 in the early months of the pandemic, the time intervals between assessments varied depending on when patients enrolled: across participants, the time difference between Timepoint 1 and 2 was 12 to 43 weeks (M = 25.1, SD: 8.44), whereas the difference between Timepoint 2 and 3 was 1 to 33 weeks (M = 12.0, SD: 8.54). The time lag was evaluated for its predictive value on EPII and pain interference scores and was found not to be a significant predictor for either measure.

### Measures

#### Demographics and study variables

In the baseline survey, participants answered questions about their socio-demographics. Specific covariates included in the current analysis were selected based on well-documented associations with pain and opioid use: biological sex at birth (male/female), racial/ethnic identity, age in years, and annual income.

#### Days of opioid use

At baseline, the Timeline Followback [34] was used to assess the number of days within the past month each participant reported any non-prescribed opioid and other substance use. For this study, we examined the number of days of reported heroin use and the number of days of reported painkiller or other analgesic use (not as prescribed). These two variables were then combined to create a composite ‘days of opioid use’ variable, which reflected use pre-pandemic and prior to MOUD induction.

#### Pain interference

At baseline and each follow-up assessment, participants self-reported pain interference on the Brief Pain Inventory [35]. Focal items inquired whether pain had interfered with seven domains (general activity, mood, walking ability, normal work, relations with other people, sleep, and enjoyment of life) during the past 24 h, on a scale from 0 (does not interfere) to 10 (completely interferes). Reliability for assessment of pain interference was very high (α = 0.95 at baseline, at 6-month follow-up, and at 9-month follow-up). We calculated a mean score representing the average extent of pain interference on activities across these seven domains.

#### EPII Survey

In order to investigate how experiences of pandemic-induced isolation impacted participants, we aggregated the number of EPII endorsed isolation-related items. This included 10 items from the “social isolation” domain (e.g., “Separated from family or close friends”; “Unable to participate in social clubs, sports teams, or usual volunteer activities”) and 3 items from the “quarantining and physical distancing” domain (“Isolated or quarantined due to possible exposure to this disease”, “Limited physical closeness with child or loved one due to concerns of infection”, and “Moved out or lived away from family due to a high-risk job [e.g., health care worker, first responder]”). Total possible scores on this isolation-related impact inventory ranged from 0 to 13. We also identified a set of four EPII items that were risk factors for pain interference (e.g., “more time sitting down or being sedentary”) which we examined as potential covariates. As the EPII signals endorsement of events, it is not advised to compute reliability statistics from this type of data [36].

### Statistical analysis

The analytical sample was determined as those who completed the EPII survey. A 71% response rate was obtained for the EPII, yielding an analytical sample of 133 respondents. Sensitivity analyses found that completers were representative of the full Project [Masked] sample, except that non-completers more often identified as male (*t*(186) = 2.790, *p* = .006).

Preliminary analyses examined whether condition (assignment to implementation strategy condition), timing of when the EPII was completed (before the 6 or 9-month follow-up), or contingency management dosage (number of sessions received) were associated with the focal items, but no significant associations were identified. Additional analyses determined that most of the sociodemographic variables (sex, race, ethnicity, and SES) were not related to pain interference. Thus, data were pooled across conditions, and none of the aforementioned variables (e.g., timing, number of sessions, sex, race, ethnicity, SES) were controlled for in analyses. The only sociodemographic variable that predicted pain interference was age, such that older participants reported greater experiences of pain interference pre-pandemic and during the pandemic (*r*s 0.201 and 0.181 respectively, *p*s < 0.05). When pre-pandemic pain interference was included as a predictor of pain interference during the pandemic, partial correlations demonstrated that the effect of age was no longer significant (partial *r* = .11, *p* = .23); hence, age was also not included as a covariate in the final analysis.

We examined associations between pre-pandemic pain interference (Timepoint 1), pandemic-induced isolation (Timepoint 2) and pain interference during the pandemic (Timepoint 3), controlling for pre-pandemic opioid use (Timepoint 1). In order to examine whether pandemic-induced isolation statistically mediated the effect of pre-pandemic pain interference on pain interference during the pandemic, we estimated a path model using Mplus version 8.6 [37]. In this model, the statistical mediator (M: pandemic-induced isolation; continuous) and outcome (Y: follow-up pain; continuous) were modeled with a continuous distribution of mean and variance, using a full-information maximum likelihood estimator. The statistical mediator was predicted by pre-pandemic pain interference (X_1_) and opioid use (X_2_), and the outcome was predicted by pre-pandemic pain interference, pre-pandemic opioid use, and pandemic-induced isolation. The indirect association between pre-pandemic pain interference and pain interference during the pandemic through pandemic-induced isolation was modeled as the bias-corrected bootstrapped product of terms [38]. Specifically, we multiplied the association of pre-pandemic pain interference and pandemic-induced isolation (a-path) and the association of pandemic-induced isolation and pain interference during the pandemic (b-path). This product was bootstrapped 5000 times and the bootstrapped bias-corrected confidence interval determined from the range of bootstrapped results, to obtain reliable confidence intervals given the known tendency of product-terms to not be normally distributed [39]. It was concluded that a significant indirect association was present if the bootstrapped confidence interval for the estimate of the indirect association did not contain zero.

As a final sensitivity analysis, we added the four EPII items to the path model that were not directly related to social isolation but were risk factors of pain interference (described in Measures). This sensitivity analysis did not significantly improve our ability to predict follow-up pain interference. We therefore focus solely on the EPII items assessing pandemic-induced social isolation and do not include these additional items in the analysis.

## Results

Table 1 contains sociodemographic and substance use information on participants from the analytical sample (*n* = 133). Briefly, participants from were predominantly Non-Hispanic White (83%), female (60%), and had completed high school (60%), with a median age of 34 years (IQR: 29, 41). The vast majority of the participants had been inducted on methadone (88%), with 11% inducted on buprenorphine and 1% inducted on naltrexone. Participants reported using non-prescribed opioids almost daily at baseline (M = 24.8, SD: 21.3, range [0:30]), whereas use of other substances was much less frequent, with the exception of cigarettes (M = 22.9, SD: 12.2, range [0:30]). On average, participants reported experiencing six pandemic-induced isolation-related experiences (on 13 items, M = 6.0, SD: 3.13, range [0:12]).

**Table 1:** Sample sociodemographic and baseline substance use characteristics (*n* = 133)

				Range	
		N (%)	M (SD)	Min	Max
Age			36 (9.8)	20	68
Race					
American Indian or Alaska Native		2 (1)			
Asian		1 (1)			
Black or African American		6 (5)			
Native Hawaiian or Other Pacific Islander		2 (1)			
White		111 (84)			
More than One Race		5 (4)			
Other		6 (5)			
Ethnicity					
Not Hispanic/Latino		120 (90)			
Hispanic/Latino		13 (10)			
Gender					
Male		54 (41)			
Female		79 (59)			
Medications for opioid use disorder (prescribed)					
Methadone		117 (88)			
Buprenorphine		15 (11)			
Naltrexone		1 (1)			
Days of substance use over the past 30 days (not as prescribed)					
Opioids			24.8 (21.3)	0	30
Marijuana (including THC and Other Cannabinoids)			6.83 (10.66)	0	30
Cocaine (excluding Crack Cocaine)			2.01 (5.33)	0	30
Crack (Smoked Rock or Freebase)			2.87 (6.60)	0	30
Amphetamine-Type Stimulants			1.94 (6.95)	0	30
Hallucinogens (including MDMA/Ecstasy)			0.06 (0.48)	0	5
Sedatives and Hypnotics (excluding Benzodiazepines)			1.5 (5.74)	0	30
Benzodiazepines			3.6 (8.26)	0	30
Inhalants			0.45 (3.68)	0	30
Cigarettes (Combustible)			22.89 (12.16)	0	30

In the baseline pre-pandemic assessment, participants reported a mean score of pain interference at 3.88 (SD: 3.04, range: [0:10]), indicating moderate interference from pain on daily activities. In the follow-up assessment during the pandemic, participants reported pain interference scores of 3.41 (SD: 2.80, range: [0:10]), indicating an overall small, but non-significant (*p* = .071) reduction in reported pain at follow-up across the sample. Table 2 shows the correlations among the model variables.

**Table 2:** Correlations among pre-pandemic opioid use (Timepoint 1), pandemic-induced isolation (Timepoint 2), and pain interference (Timepoints 1 and 3; *n* = 133)

	Timepoint 1		Timepoint 2	Timepoint 3
	*Pain Interference*	*Days of Opioid Use*	*Pandemic-Induced Isolation*	*Pain Interference*
**Timepoint 1: Pre-Pandemic** *August 2019 – March 2020*				
Pain Interference	---			
Days of Opioid Use	0.107	---		
**Timepoint 2: During Pandemic** *March 2020 – May 2020*				
Pandemic-Induced Isolation	0.211*	0.229**	---	
**Timepoint 3: During Pandemic** *May 2020 – January 2020*				
Pain Interference	0.417***	− 0.011	0.218*	---

Note: Timepoint 1 occurred prior to patients’ induction on medication for opioid use disorder (MOUD). Timepoints 2 and 3 occurred after the patient had been inducted and while they were actively receiving MOUD; *p < .05, **p < .01, ***p < .001

**Fig. 1:**
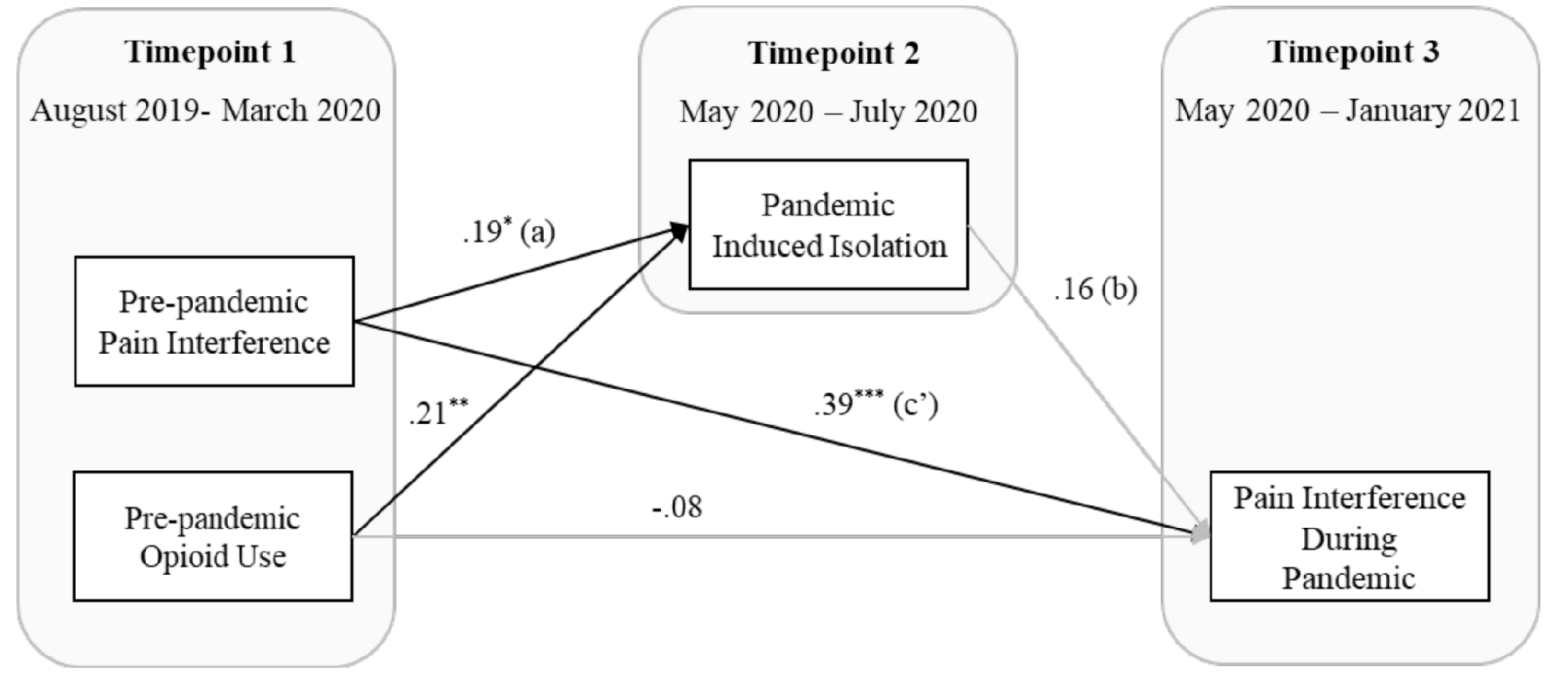
Path model of relations between pre-pandemic pain interference, pre-pandemic opioid use, pandemic induced isolation, and pain interference during pandemic (*n* = 133). Coefficients represent standardized effects. *p <0.05, **p <0.01, ***p <0.001. Bootstrapped product of a-path and b-path was significant at 95% CI: B=0.03, 95% bCI: [0.01 - 0.09]

Figure 1 depicts the model results from the path model investigating the association between pre-pandemic pain interference and pain interference during the pandemic via pandemic-induced isolation, while controlling for pre-pandemic opioid use. Results of the path model indicated that both pre-pandemic pain interference and opioid use at Timepoint 1 were positively and significantly related to pandemic-induced isolation at Timepoint 2. Pre-pandemic opioid use at Timepoint 1 was negatively related to pain interference during the pandemic (i.e., more opioid use at Timepoint 1, less pain interference at Timepoint 3), however this path was not statistically significant. When examining the path between pandemic-induced isolation (Timepoint 2) and pain interference during the pandemic (Timepoint 3), the effect was positive, but not significant (β = 0.16, *p* = .08).

Importantly, results from the bootstrapped estimation of the indirect relation between baseline pain interference and pain interference during the pandemic via pandemic-induced isolation revealed a significant indirect effect (β = 0.03, 95% CI: [0.01–0.09]. Consistent with hypotheses, the bias-corrected bootstrapped product of terms [38] describing the association of pre-pandemic pain to pandemic-induced isolation (a-path; Timepoint 1 to Timepoint 2) and the association of pandemic-induced isolation to pain interference during the pandemic (b-path; Timepoint 2 to Timepoint 3) revealed a significant, positive effect. Hence, pandemic-induced isolation (Timepoint 2) partially mediated the effect of pre-pandemic pain interference (Timepoint 1) on pain interference experienced during the pandemic (Timepoint 3).

## Discussion

The current study conducted a secondary data analysis examining whether social isolation during COVID-19 may have partially explained changes in pain interference among patients on MOUD. Controlling for pre-pandemic opioid use, we hypothesized that social isolation during COVID-19 would partially mediate the effect of pre-pandemic pain interference on pain interference during the pandemic. Consistent with this hypothesis, we found that pre-pandemic pain interference had a significant indirect effect on pain interference during the pandemic through pandemic-induced isolation. COVID-19 led to an unprecedented shift to take-home MOUD and remote treatment provision, as well as a shift to remote work and social engagement, reducing opportunities for in-person interactions and social connections [40, 41]. Our analysis examined the extent to which pandemic-induced isolation affected subsequent pain interference. Specifically, higher levels of pre-pandemic pain interference were significantly related with levels of pain interference six- to nine-months later, and these pain levels were partially explained by the level of pandemic-induced isolation reported. These findings can be contextualized within the broader literature examining the impacts of chronic pain on daily life. Previous research has shown that higher pain levels significantly disrupt employment [42] and social interactions [27], resulting in heightened loneliness and isolation [26]. Similarly, social isolation has been posited to reduce motivation to seek preventative care, adhere to medication, and practice self-care [43]. These existing challenges were likely exacerbated during the pandemic, contributing to higher levels of social isolation. We also found that both pre-pandemic opioid use and pain interference were associated with higher levels of pandemic-induced social isolation. Given the well-documented spikes in both social isolation and overdose-related deaths in the early months of the pandemic, our results suggest that those patients who would most benefit from social connection – patients with the highest use of opioids and the highest rates of pain – were the most likely to report feeling disconnected and isolated during COVID-related social distancing and that this, in turn, partially explained changes in levels of pain interference. Consistent with previous research, these findings may be related to marginalization or stigma associated with both chronic pain and/or SUD [3, 27, 44, 45]. Past research has shown that discrimination experienced by underserved populations can impair immune functioning [44, 46, 47], compounding the negative effects of chronic pain [45] and SUD stigma [48].

Interestingly, there were no sex differences in pandemic-reported social isolation, even though women are consistently found to report higher levels of both pain and social isolation [49–51]. Similarly, older age was not related to pandemic-induced isolation, counter to previous research [52], but was related to higher pain interference levels consistent with prior research findings [53]. One possible reason for the lack of significant effects of biological sex and age may be that the entire United States population was experiencing unprecedented levels of social isolation, thereby limiting our ability to detect effects of socio-demographic variables that typically predict social interactions.

Another unanticipated finding was that the level of pain interference experienced by this sample of patients on MOUD was lower at the assessment during the pandemic, compared to the pre-pandemic assessment. This finding was at odds with the results of epidemiological studies that found that non-prescription opioid use can predict negative perceived general health prospectively [54] and pain interference levels increased among the general population and among patients with chronic pain [24–26], during COVID-related social distancing. It is possible that our results are affected by the timing of patient enrollment in the parent study. Patients were enrolled into Project [Masked] shortly after their induction on MOUD, and even though pain is associated with worse MOUD outcomes [55], the receipt of MOUD with methadone or buprenorphine has been shown to effectively reduce pain [56, 57]. The fact that we were able to detect mediation effects of pandemic-induced social isolation on changes in pain interference, despite an overall reduction in pain interference levels, raises confidence in the stability of the effects.

Although novel, these findings come with limitations. First, although we did not detect systematic differences between survey respondents and non-respondents, it is possible that participants who were not able to complete the survey due to limited telephone or internet access might reflect a subgroup of individuals who experienced greater levels of pandemic-induced social isolation. Second, this study was non-experimental (e.g., no random assignment to pain) and therefore we cannot make causal assertions about the observed effects. We can simply conclude that pandemic-induced social isolation partially mediated changes in pain interference during the pandemic. Third, we studied the effects of pandemic-induced social isolation and pain because these are known risk factors for overdose, but we did not track the actual rate of overdose in the sample. Finally, the analytical sample was drawn from eight opioid treatment programs (OTPs) in the New England region of the United States and should not be considered representative of OTPs throughout the United States.

Despite these limitations, this study is among the first to show that pain interference prior to the pandemic predicted pandemic-induced isolation, and that this isolation in turn partially explained pain among patients on MOUD. Our path model further strengthens the argument for a bidirectional relationship between pain and social isolation. Understanding the relationship between social isolation and pain interference among patients with MOUD is important, given that pain is a well-established predictor of OUD, returning to opioid use, and worse treatment outcomes among patients receiving MOUD. Most importantly, this study highlights social isolation as a key risk factor for patients on MOUD and suggests that interventions promoting social connection could be associated with reduced pain interference, which in turn could predict greater quality of life. Future research would benefit from examining social isolation among a larger group of patients on MOUD to further explore as the complex, and likely bi-directional relationship between pain interference and social isolation.

## Conclusion

In summary, this study is among the first to demonstrate that social isolation during the early months of the COVID-19 pandemic partially explained pain interference among patients on MOUD, even when controlling for opioid use and pain interference at baseline. The current findings have at least two key clinical implications. First, given the high rates of pain interference and social isolation reported in this study, paired with the fact that MOUD patients are already less likely to seek medical care due to stigma and reports of substandard care [3] programs that serve MOUD patients would likely benefit from routine assessments of pain and isolation. Such assessments could guide referrals to ancillary services and potentially improve the treatment outcomes of patients on MOUD. Second, programs that provide MOUD could consider offering services such as connection to a peer recovery counselor to foster a sense of belonging or community to try and reduce patients’ social isolation. As we continue identifying the lingering harms of COVID-19 beyond physical illness, it is important to consider emotional effects such as social isolation and strategies to mitigate these harms: our results suggest that services targeting social connection may be particularly beneficial for MOUD patients.

## Data Availability

The data generated and analyzed during the current study are not publicly available due to ongoing study procedures but are available from the corresponding author upon reasonable request.

## Abbreviations

US: United States
SUD: Substance use disorder
OUD: Opioid use disorder
MOUD: Medications for opioid use disorder
OTPs: Opioid treatment programs
IRB: Institutional Review Board
